# Acute Kidney Injury and Early Predictive Factors in COVID-19 Patients

**DOI:** 10.3389/fmed.2021.604242

**Published:** 2021-07-12

**Authors:** Jiaye Liu, Tingyan Wang, Qingxian Cai, Deliang Huang, Liqin Sun, Qing He, Fu-Sheng Wang, Jun Chen

**Affiliations:** ^1^National Clinical Research Center for Infectious Diseases, The Third People's Hospital of Shenzhen, The Second Affiliated Hospital of Southern University of Science and Technology, Shenzhen, China; ^2^Nuffield Department of Medicine, University of Oxford, Oxford, United Kingdom; ^3^Treatment and Research Center for Infectious Diseases, The Fifth Medical Center of PLA General Hospital, Beijing, China

**Keywords:** coronavirus, COVID-19, SARS-CoV-2, creatinine, eGFR, acute kidney injury

## Abstract

**Objectives:** Our objective was to explore the incidence and early predictive factors of acute kidney injury in coronavirus disease 2019 (COVID-19) patients.

**Method:** We established a retrospective cohort of 408 patients who were admitted to Shenzhen Third People's Hospital in Shenzhen, China, between January 1 and March 31, 2020. Clinical outcomes and renal function were monitored until April 12, 2020, with a median follow-up duration of 21 days [interquartile range (IQR) = 14–33].

**Results:** When first admitted to hospital (baseline), 19.36% (79/408) presented renal dysfunction [estimated glomerular filtration rate (eGFR) <90 ml/min/1.73 m^2^]. During follow-up, 3.9% (16/408) developed acute kidney injury (AKI). Age ≥60 years [hazard ratio (HR) = 4.78, 95% CI = 1.10–20.69], PaO_2_/FiO_2_ ratio <300 (HR = 3.48, 95% CI = 1.04–11.62), and higher creatinine (HR = 1.04, 95% CI = 1.01–1.07) at baseline independently predicted the risk of AKI. Respectively, 25.0% (102/408), 3.9% (16/408), 0.5% (2/408), 1.0% (4/408), and 0.2% (1/408) experienced G2, G3a, G3b, G4, and G5 as their most severe category during hospitalization, while 69.4% (283/408) had normal eGFRs throughout the follow-up period. When finally discharged from hospital, there were 12.5% (51/408) of patients with abnormal eGFRs.

**Conclusions:** COVID-19 patients can be at risk of AKI and continuous eGFR decline during hospitalization, which can be early predicted by baseline factors. Some individuals still had renal dysfunction when finally discharged from hospital.

## Background

Coronavirus disease 2019 (COVID-19) is an emerging respiratory disease caused by severe acute respiratory syndrome coronavirus 2 (SARS-CoV-2). According to the updated information of the “Coronavirus disease 2019 (COVID-19) Situation Report” by WHO, a total of 169,604,858 confirmed cases and 3,530,837 deaths were reported globally as of May 30, 2021 (1). Unfortunately, targeted drugs have not been available to date, and the number of infections is still growing worldwide. For the foreseeable future, COVID-19 could constantly pose a great threat to human health.

Most of the published articles on COVID-19 highlighted the lungs as the main organ involved in the disease (2–4), and a series of studies have also reported data regarding injury in the liver (5–7), the cardiovascular system (8, 9), and the gastrointestinal tract (10). Unfortunately, a few studies have also reported an increased incidence of renal injury following diagnosis of COVID-19, e.g., acute kidney injury (AKI) in COVID-19 patients varying from 0.1 to 29% (11–15). Meanwhile, a large cohort study from China revealed that 44% of COVID-19 patients developed proteinuria or hematuria, 15.5% had an increase of blood creatinine, and 14.1% presented an increase of blood urea nitrogen; these kidney dysfunction-related events were identified as independent associated factors for mortality (14). Another study on renal histopathological analysis of 26 autopsies of patients with COVID-19 reported that immunostaining with a SARS-CoV nucleoprotein antibody was positive in the tubule epithelium (16), which provided direct evidence of the invasion of SARS-CoV-2 into the kidney tissue. In addition, systemic hypoxia, abnormal coagulation, and possible drug or hyperventilation-relevant rhabdomyolysis could also contribute to kidney injury in COVID-19 patients (17, 18).

In summary, the existing studies mainly reported various incidences of AKI or described abnormalities in kidney laboratory tests using cross-sectional data; however, the longitudinal changes of renal function and the early predictive factors of renal dysfunction in COVID-19 patients have not been well characterized and evaluated yet. Therefore, it is quite urgent to add longitudinal data regarding renal function in COVID-19 patients. In this study, we aimed to investigate the dynamics of renal function and the risk of AKI in COVID-19 patients during hospitalization and explore the predictive factors in the early stage.

## Methods

### Study Design and Data Collection

All confirmed COVID-19 patients admitted to Shenzhen Third People's Hospital between January 1 and March 31, 2020, were enrolled. Shenzhen Third People's Hospital, located in Guangdong, China, is the designated hospital with the largest number of COVID-19 cases outside Hubei in China. All the subjects with COVID-19 enrolled in this study were diagnosed according to the WHO interim guidance. The clinical outcomes of COVID-19 and renal function were monitored up to April 12, 2020. During the follow-up period, patients may include one or more hospital admission(s), e.g., there were 43 patients who were re-admitted to the hospital due to recurrence of positive SARS-CoV-2. The inclusion criteria were: (1) subjects diagnosed with COVID-19; (2) subjects with the records well-documented; and (3) subjects with longitudinal follow-up, i.e., renal function testing [i.e., estimated glomerular filtration rate (eGFR) and creatinine] with at least across 2 days during follow-up. Subjects with missing data at baseline for eGFR, creatinine, or severity of COVID-19 were excluded. Finally, 408 COVID-19 patients who met the above eligibility criteria were selected for analysis in this study.

The study protocol was consistent with the ethical guidelines of the 1975 Declaration of Helsinki and was approved by the Institutional Review Board of Shenzhen Third People's Hospital (2020-183). All subjects provided signed informed consent.

### Confirmation of COVID-19

The presence of SARS-CoV-2 was detected using the real-time reverse transcription polymerase chain reaction (RT-PCR) method. Two pairs of primers targeting the open reading frame 1ab (ORF1ab) and the nucleocapsid protein (N) were amplified and examined. Each sample was run in triplicate with a positive and negative control set, as suggested. These diagnostic criteria were based on the recommendations of the Chinese Center for Disease Control and Prevention (CDC). Samples identified as positive for SARS-CoV-2 by the local laboratory, further confirmed by the Key Laboratory of Shenzhen CDC, China.

### Clinical Evaluation, Follow Up, and Outcomes

Baseline was defined as the first hospital admission due to COVID-19. At baseline and during follow-up, all subjects included in this study underwent routine examination, monitoring of renal function, and SARS-CoV-2 nucleic acid testing with a median interval of 3 days. The median follow-up period of patients was 21 days (IQR = 14–33).

Serum creatinine was used for assessing AKI-related events and eGFR was used for monitoring whether a decline occurs in renal function during follow-up. The eGFRs were classified into six categories according to the Kidney Disease Improving Global Outcomes (KDIGO) guideline (19): G1: ≥90 ml/min/1.73 m^2^ (normal); G2: 60–89 ml/min/1.73 m^2^ (mildly decreased kidney function); G3a: 45–59 ml/min/1.73 m^2^ (mild-moderate loss of kidney function); G3b: 30–44 ml/min/1.73 m^2^ (moderate–severe loss of kidney function); G4: 15–29 ml/min/1.73 m^2^ (severe loss of kidney function); and G5: <15 ml/min/1.73 m^2^ (kidney failure). Abnormal eGFR is defined as <90 ml/min/1.73 m^2^, i.e., for which the eGFR is at a category of G2, G3a, G3b, G4, or G5. Urine tests were used to assess the proportion of proteinuria or hematuria for patients at baseline.

The main outcome was the incidence of AKI, which was defined as an elevation in serum creatinine of ≥0.3 mg/dl (26.5 μmol/L) or ≥1.5 times compared to a previous time point. The time to event was defined as the period from the date of the first hospital admission to the date of occurrence of the defined outcome. Patients were censored at death, discharge, or the last follow-up visit.

### Statistical Analysis

All analyses were carried out using R software version 3.6.1. Firstly, we described the baseline characteristics for all subjects and for subgroups stratified by baseline renal function (eGFR <90 vs. ≥90 ml/min/1.73 m^2^).

Secondly, we calculated the total proportions of AKI occurrence during follow-up and then summarized these incidences stratified by baseline characteristics (age, sex, and eGFR), using χ^2^ test or Fisher's exact test to compare the proportions between subgroups. Furthermore, we performed Cox regression analysis to examine the early predictive factors of an AKI event. Covariates were included in the multivariable model if they had a *p*-value < 0.1 in the univariable Cox regression analysis, or if they were considered to be key factors from the clinical perspective.

Thirdly, we analyzed the dynamics of eGFR during follow-up. Specifically, we investigated the most severe renal dysfunction of each individual during follow-up and summarized the proportion of patients who still had abnormal eGFRs when finally discharged from the hospital.

All significance tests performed were two-sided. Values of *p* < 0.05 were deemed statistically significant, and 95% confidence intervals (CIs) were calculated for point estimates.

## Results

### Baseline Characteristics

The baseline characteristics of the subjects are summarized in Table 1. At baseline, 19.36% (79/408) presented eGFR <90 ml/min/1.73 m^2^ at baseline. In the subgroup of patients who had renal dysfunction at baseline, the eGFR categories G2, G3a, G3b, and G4 accounted for 86.1% (68/79), 11.4% (9/79), 1.3% (1/79), and 1.3% (1/79), respectively, but no patient had renal failure (G5) at baseline. Compared to those who had normal renal function at baseline, patients with renal dysfunction at baseline had higher median values of age (62 vs. 41 years), BMI (24.4 vs. 22.8 kg/m^2^), chest computed tomography (CT) score (14 vs. 9.5), C-reactive protein (CRP) (17.07 vs. 7.04 mg/L), urea (5.09 vs. 3.71 mmol/L), creatinine (89 vs. 59 μmol/L), and erythrocyte sedimentation rate (ESR) (42 vs. 25 mm/h), while they had lower median values of the PaO_2_/FiO_2_ ratio (P/F ratio) (368.57 vs. 432.38), platelet count (152 vs. 192 × 10^9^/L), and lymphocyte (1.13 vs. 1.36 × 10^9^/L) (all *p* < 0.05). Meanwhile, the renal dysfunction subgroup had a higher proportion of males (68.4% vs. 43.2%), a higher percentage of severe COVID-19 patients at baseline (13.9% vs. 3.0%), and a higher proportion of individuals who had more than one comorbidity (50.6% vs. 34%), compared to the group with normal renal function at baseline (all *p* < 0.05). Before hospital admission due to COVID-19, 65/408 (15.9%) patients took a long-term course of medicines for comorbidities (Supplementary Table 1). None of patients had chronic kidney disease (CKD) before hospital admission according to medical history records. Additionally, 389 out of 408 patients had urine dipstick at baseline. Urine protein was negative in 84.32% (328/389), while positive with +2 to +3 in 2.05% (8/389). Hematuria was negative in 89.46% (348/389), but positive with +2 to +3 in 4.39% (17/389) (Supplementary Table 2).

**Table 1:** Baseline characteristics of the selected COVID-19 patients for this study.

	**Overall**	**Normal renal function at baseline**	**Renal dysfunction at baseline**	***p*-value**
Number of patients	408	329	79	
Age, median (IQR) (years)	47 (34–60)	41 (32–56)	62 (55–69)	<0.001
Age ≥60 years	104 (25.5)	57 (17.3)	47 (59.5)	<0.001
Male	196 (48.0)	142 (43.2)	54 (68.4)	<0.001
BMI, median (IQR) (kg/m^2^)	23.0 (21.2–25.6)	22.8 (20.8–25.2)	24.4 (22.0–26.6)	0.002
**Case severity**				<0.001
Mild	43 (10.5)	39 (11.9)	4 (5.1)	
Moderate	344 (84.3)	280 (85.1)	64 (81.0)	
Severe	19 (4.7)	10 (3.0)	9 (11.4)	
Critical	2 (0.5)	0 (0.0)	2 (2.5)	
Time from illness onset to admission, median (IQR) (days)	3 (1–6)	3 (1–6)	3 (2–6)	0.574
**Number of comorbidities**				<0.001
0	289 (70.8)	250 (76.0)	39 (49.4)	
1	88 (21.6)	67 (20.4)	21 (26.6)	
2	23 (5.6)	8 (2.4)	15 (19.0)	
3	8 (2.0)	4 (1.2)	4 (5.1)	
**Comorbidity types**				
Diabetes	22 (5.4)	12 (3.6)	10 (12.7)	0.004
Hypertension	58 (14.2)	35 (10.6)	23 (29.1)	<0.001
Cardiovascular disease	35 (8.6)	15 (4.6)	20 (25.3)	<0.001
Cancer	5 (1.2)	3 (0.9)	2 (2.5)	0.545
Chronic liver disease	38 (9.3)	30 (9.1)	8 (10.1)	0.951
P/F ratio, median (IQR)	420.48 (356.67–476.02)	432.38 (372.38–485.71)	368.57 (317.26–406.90)	<0.001
CT score, median (IQR)	10.00 (4.00–16.00)	9.50 (2.75–15.00)	14.00 (10.00–22.000	<0.001
ALT, median (IQR) (U/L)	21.00 (15.00–31.00)	20.00 (14.00–29.50)	25.30 (19.00–34.70)	0.001
AST, median (IQR) (U/L)	26.10 (21.00–35.52)	25.00 (20.00–33.90)	31.00 (24.75–42.10)	<0.001
TBIL, median (IQR) (μmol/L)	10.90 (8.30–16.15)	10.70 (8.20–15.40)	12.60 (8.70–21.55)	0.015
GGT, median (IQR) (U/L)	23.30 (16.00–36.25)	22.00 (15.00–35.00)	30.00 (20.90–40.00)	0.003
Fibrinogen, median (IQR) (g/L)	3.84 (3.08–4.60)	3.67 (3.03–4.52)	4.27 (3.75–5.04)	<0.001
Platelet count, median (IQR) (×10^9^/L)	186.00 (148.75–230.25)	192.00 (154.00–236.00)	152.00 (131.50–190.00)	<0.001
Lymphocyte count, median (IQR) (×10^9^/L)	1.31 (0.99–1.80)	1.36 (1.04–1.91)	1.13 (0.88–1.54)	0.001
C-reactive protein, median (IQR) (mg/L)	8.75 (3.43–24.77)	7.04 (2.64–22.23)	17.07 (8.66–37.37)	<0.001
Fibrosis-4, median (IQR)	1.38 (0.80–2.37)	1.27 (0.73–1.85)	2.67 (1.66–4.00)	<0.001
AST-to-platelet ratio index, median (IQR)	0.32 (0.23–0.48)	0.30 (0.22–0.43)	0.46 (0.33–0.63)	<0.001
eGFR, median (IQR) (ml/min/1.73 m^2^)	105.18 (93.44–115.82)	109.38 (99.81–118.84)	77.88 (67.88–84.97)	<0.001
**eGFR categories (ml/min/1.73 m**^**2**^**)**				<0.001
G1: ≥90	329 (80.6)	329 (100.0)	0 (0.0)	
G2: 60–89	68 (16.7)	0 (0.0)	68 (86.1)	
G3a: 45–59	9 (2.2)	0 (0.0)	9 (11.4)	
G3b: 30–44	1 (0.2)	0 (0.0)	1 (1.3)	
G4: 15–29	1 (0.2)	0 (0.0)	1 (1.3)	
Urea, median (IQR) (mmol/L)	3.92 (3.21–4.81)	3.71 (3.10–4.42)	5.09 (4.25–5.90)	<0.001
Creatinine, median (IQR) (μmol/L)	63.00 (53.00–77.00)	59.00 (50.00–72.00)	89.00 (74.00–99.50)	<0.001
Urea/creatinine, median (IQR)	60.73 (49.23–74.97)	61.29 (49.57–78.94)	58.39 (47.67–67.31)	0.065
ESR, median (IQR) (mm/h)	28 (14–48)	25 (14–45)	42 (25–63)	<0.001
Fever	274 (67.2)	215 (65.3)	59 (74.7)	0.146

*BMI, body mass index; ALT, alanine aminotransferase; AST, aspartate aminotransferase; TBIL, total bilirubin; GGT, γ-glutamyl transpeptidase; eGFR, estimated glomerular filtration rate; ESR, erythrocyte sedimentation rate; P/F ratio, PaO_2_/FiO_2_ ratio; IQR, interquartile range*.

### AKI Occurrence During Follow-Up

Overall, 3.9% (16/408) developed AKI during follow-up (Table 2). With a subgroup analysis by baseline characteristics, we observed that patients aged ≥60 years, with at least one comorbidity, or with severe COVID-19 at baseline had a higher incidence of AKI events compared to the control groups, i.e., 10.6% in the group aged ≥60 years vs. 1.6% in the group aged <60 years (*p* < 0.001), 7.6% in patients who had at least one comorbidity vs. 2.4% in those without comorbidity (*p* = 0.031), and 14.3% in the baseline severe COVID-19 group vs. 3.4% in the baseline non-severe COVID-19 group (*p* = 0.043). Most of the drugs used before COVID-19 were antihypertensives and hypoglycemics, which are not nephrotoxic and less likely to cause AKI. Although three patients took steroids, none of them experienced AKI during hospitalization. We then summarized the occurrences of AKI stratified by medicine use during hospitalization (Supplementary Table 3). Patients who took steroids during hospitalization had a higher incidence of AKI events compared to those who did not take steroids (9.1 vs. 2.0%, *p* = 0.003). Similarly, the incidence of AKI was 8.6% in those taking antibiotics vs. 1.5% in those without using antibiotics (*p* <0.001) (Supplementary Table 3).

**Table 2:** Total proportions of AKI occurrence during follow-up stratified by baseline characteristics.

**Stratifying variables at baseline**	**Subgroups**		**Patients without AKI events**	**Patients with an occurrence of AKI event**	**Proportion**	***p*-value**
Overall			392	16	16/408 (3.9%)	
Age at baseline	Age <60 years	*n* = 304	299	5	5/304 (1.6%)	0.0002
	Age ≥60 years	*n* = 104	93	11	11/104 (10.6%)	
Baseline eGFR	Baseline eGFR ≥90	*n* = 329	319	10	10/329 (3.0%)	0.121
	Baseline eGFR <90	*n* = 79	73	6	6/79 (7.6%)	
No. of comorbidities^a^	None	*n* = 289	282	7	7/289 (2.4%)	0.031
	At least one	*n* = 119	110	9	9/119 (7.6%)	
Severity of COVID-19 at baseline	Non-severe	*n* = 387	374	13	13/387 (3.4%)	0.043
	Severe	*n* = 21	18	3	3/21 (14.3%)	

*eGFR, estimated glomerular filtration rate*.

a *Types of comorbidities: hypertension, cardiovascular disease, diabetes, liver diseases, and cancer*.

### Risk Factors to Early Predict AKI

**Figure 1:**
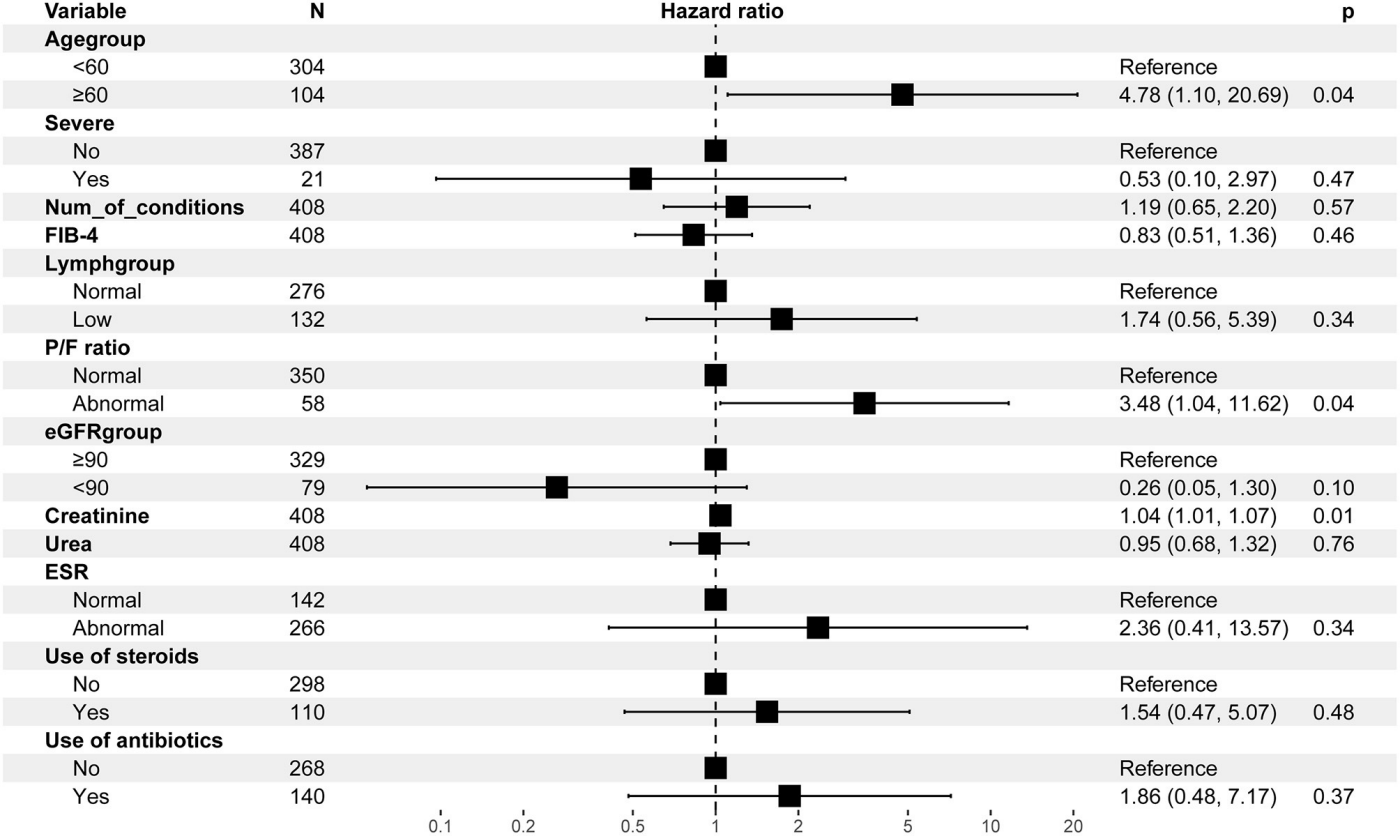
Multivariable Cox regression analysis for the risk of AKI-related events. AKI, acute kidney injury; FIB-4, fibrosis-4; ESR, erythrocyte sedimentation rate; P/F ratio, PaO_2_/FiO_2_ ratio. 43 observations were not included in the multivariable analysis due to missing data.

The multivariable Cox regression analysis showed that age ≥60 years [hazard ratio (HR) = 4.78, 95% CI = 1.10–20.69], a P/F ratio <300 (HR = 3.48, 95% CI = 1.04–11.62), and a higher creatinine (HR = 1.04, 95% CI = 1.01–1.07) at baseline independently predict the risk of AKI during follow-up (Figure 1).

### Dynamics of eGFR During Follow-up

#### Most Severe Renal Dysfunction of Each Individual During Follow-Up

**Figure 2:**
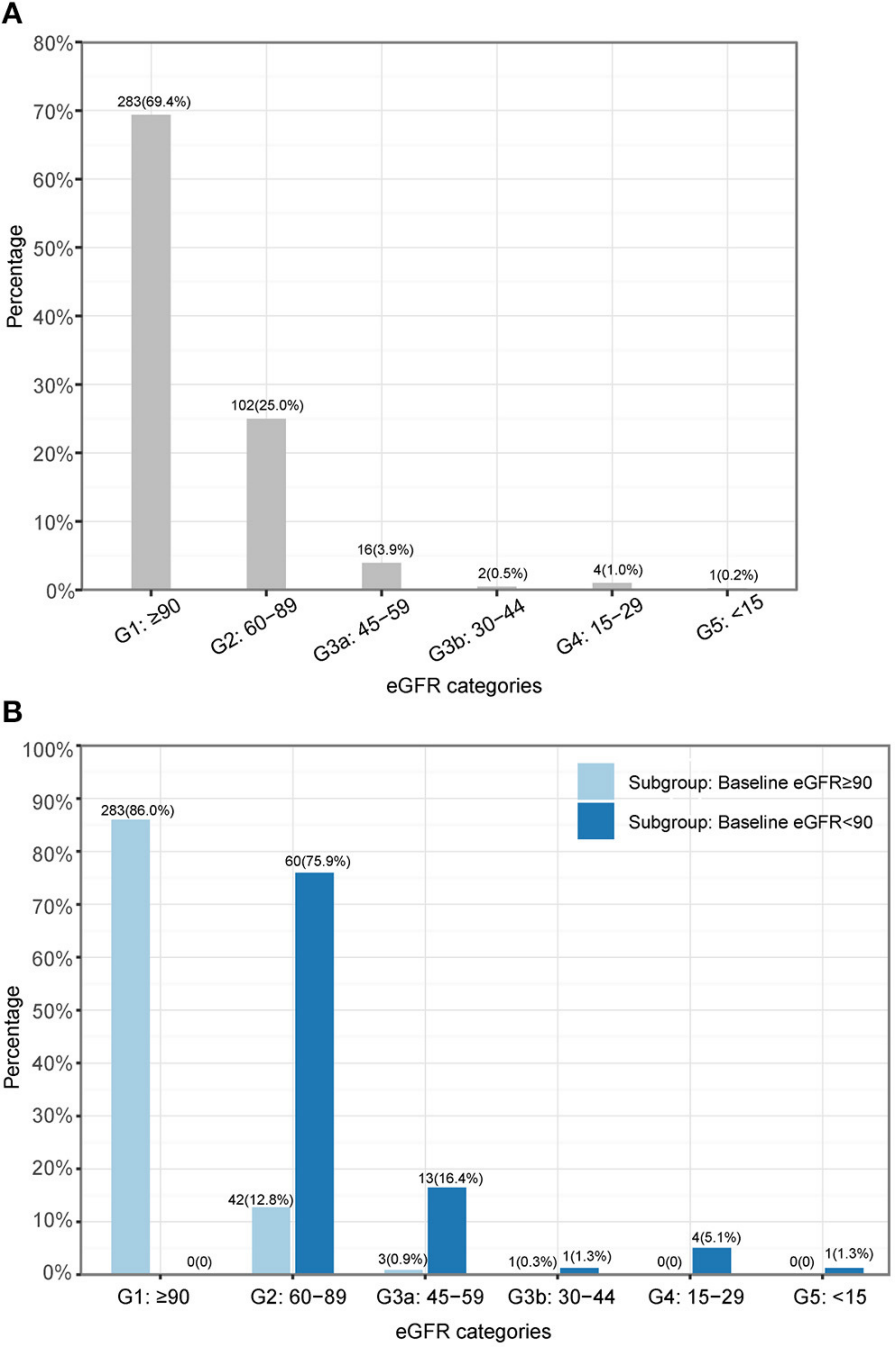
The most severe renal dysfunction of each individual during follow-up. (**A**) Overall. (**B**) Subgroups. *eGFR*, estimated glomerular filtration rate.

We observed that 25.0% (102/408), 3.9% (16/408), 0.5% (2/408), 1.0% (4/408), and 0.2% (1/408) respectively experienced G2, G3a, G3b, G4, and G5 as their most severe category during follow-up, although 69.4% (283/408) of patients had their eGFRs maintained at normal levels (i.e., eGFR category maintained at G1) throughout the follow-up period (Figure 2A). For those patients (*n* = 125) who had abnormal eGFRs during follow-up, 63.2% (79/125) had renal dysfunction at baseline. We observed that five, three, two, three, and one patient(s) in each most severe category (G2, G3a, G3b, G4, and G5) experienced AKI during hospitalization, respectively (Supplementary Table 4). In the subgroup with normal renal function at baseline, 12.8, 0.9, and 0.3% of patients respectively had G2, G3a, and G3b as the most severe eGFR category during follow-up (Figure 2B). In contrast, for the subgroup with renal dysfunction at baseline, 75.9, 16.4, 1.3, 5.1, and 1.3% respectively had G2, G3a, G3b, G4, and G5 as their most severe eGFR category during follow-up (Figure 2B).

#### Proportion of Patients With Abnormal eGFR When Finally Discharged From Hospital

At the end of follow-up, 87.50% (357/408) of patients had normal renal function (stayed at G1), while 11.76% (48/408), 0.49% (2/408), and 0.25% (1/408) were at G2, G3a, and G3b categories, respectively, and no patient had eGFR <30 when discharged from the hospital. For those patients (*n* = 51) who had abnormal eGFRs at the end of follow-up, the median [Q1, Q3] values of eGFR at baseline and at the end of follow-up, as well as the lowest eGFR during hospitalization, were 72.4 [65.4, 85.0], 79.0 [71.6, 83.5], and 67.4 [58.4, 74.9] ml/min/1.73 m^2^, respectively; 80.4% (41/51) of patients had renal dysfunction at baseline. In other words, 48.1% (38/79) of patients with abnormal eGFR at baseline had recovered before discharged from the hospital; among of them, 4 of 38 (10.5%) patients experienced AKI during hospitalization. Moreover, at the end of follow-up, seven had a deteriorating eGFR category compared to their baseline eGFR category; their median [Q1, Q3] values of eGFR at baseline and at the end of follow-up, as well as the lowest eGFR during hospitalization, were 92.2 [91.7, 96.3], 84.3 [79.8, 86.4], and 74.6 [63.3, 82.0] ml/min/1.73 m^2^, respectively.

#### Renal Function of Patients With Readmission

For the 43 patients who were re-admitted to hospital due to the recurrence of positive SARS-CoV-2, nine (20.9%), eight (18.6%), six (13.9%), and three (7.0%) patients had abnormal renal function at the first hospital admission, at the first discharge, at re-admission, and at the second discharge, respectively (Supplementary Table 5).

## Discussion

Based on a retrospective cohort study of 408 patients, this study reveals that COVID-19 patients can be at risk of AKI; moreover, a large proportion of patients still had abnormal eGFRs when finally discharged from the hospital. Furthermore, this study found that age ≥60 years, a low P/F ratio (<300), and a higher creatinine at baseline independently predict the risk of AKI. This study added more evidence concerning the longitudinal changes of renal function in COVID-19 patients and provided important information to support the management of COVID-19.

Since SARS-CoV-2 is considered to predominantly enter alveolar epithelial cells with angiotensin-converting enzyme 2 (ACE2) as its receptor, the lungs become the most severely damaged organ (11, 13). Unfortunately, a few studies have provided evidence of SARS-CoV-2 invading the kidney tissue (16, 20). In our study, we observed that the occurrence rate of AKI was 3.9%, which was similar to the pooled incidence rate of AKI (3%) in COVID-19 patients from a meta-analysis (21). Furthermore, we found that the incidence rate of AKI was significantly higher in subgroups with age ≥60 years, or who had at least one comorbidity, or with severe COVID-19 at baseline, although the overall incidence of AKI was relatively low in the whole cohort. This implied that physicians should pay more attention to these special patients, and it is quite essential to frequently monitor the renal function of these patients. It had been confirmed in previous studies that even a small rise in creatinine, a key parameter of defining AKI, is independently associated with an increased mortality in non-COVID-19 inpatients (22); recently, a study also found that AKI was associated with a higher risk of in-hospital mortality in patients with COVID-19 (23). In this study, we could not investigate the association of AKI with mortality as few (a total of three) patients died in our study. However, it is important to investigate the predictive factors in order to identify the risk of AKI as early as possible in clinical practice. This study found that age ≥60 years, P/F ratio <300, and a higher creatinine at baseline independently predict the risk of AKI. In addition to the direct effect of SAS-CoV-2 on the kidney, a lung–kidney crosstalk might be another important mechanism that causes AKI. A recent study has reported that 68% of 357 patients with acute respiratory distress syndrome (ARDS) developed AKI (24). Cytokine overproduction in lung–kidney bidirectional damage and the injury of tubular cells secondary to renal medullary hypoxia caused by ARDS could be the potential reasons of the high risk of AKI in patients with ARDS (25). The P/F ratio is commonly used to determine the onset or the severity of acute lung injury (ALI) and ARDS. Therefore, a lung–kidney crosstalk could explain the independent predictive value of a low P/F ratio (<300) on the incidence of AKI.

Typically, persistence of abnormal eGFR for >3 months is one criterion used to determine CKD (19). In this study, we could not determine whether patients had CKD as the longest follow-up duration of our study cohort was <3 months. However, a decline of the eGFR is the precondition of potential CKD (22). Thus, we investigated the dynamics of eGFR to reflect the influence of COVID-19 on the renal function. We found that 19.36% of patients with COVID-19 had abnormal eGFRs at baseline. Although the overall dynamic of eGFR presented a trend of restoration, we observed that 13.97% of patients had an eGFR decline by one or more categories during follow-up. Moreover, at the end of follow-up, 12.5% of patients still had abnormal eGFRs; among these patients, seven had a deteriorating eGFR category compared to their baseline eGFR category. This implied that not all the renal dysfunction caused by COVID-19 was transient. It is essential to monitor the renal function of all COVID-19 patients during the hospitalization and to provide continuous attention on the risk of CKD for those patients who still had abnormal eGFRs even when they have been discharged from the hospital.

The evidence described in the KDIGO guideline suggests that a moderate decline of eGFR (<25%) is also associated with an increased risk of all-cause mortality and end-stage renal disease (ESRD) (26, 27). Thus, it is crucial to investigate the most severe category of eGFR of patients during follow-up both for assessing kidney impairments caused by COVID-19 and for predicting potential long-term severe outcomes. When examining the most severe renal dysfunction during follow-up for each individual, we found that only 69.4% had maintained a normal eGFR through follow-up; however, a total of 30.6% of patients experienced an abnormality in their eGFRs. Furthermore, it was observed that 1.2% experienced more severe than the G2 category as their most severe renal dysfunction in the subgroup with normal baseline eGFR. In contrast, this was significantly higher (i.e., increasing to 24.1% from the baseline 13.9%) in the subgroup with abnormal baseline eGFR. Our results suggested that patients with baseline renal dysfunction had a higher risk of progression to more severe kidney impairments (10.1% vs. 1.2%). Therefore, the evaluation of renal function in patients with COVID-19 at presentation is essential for identifying an already occurring kidney impairment early. More importantly, intensive monitoring of renal function is crucial for COVID-19 patients who had kidney impairments when admitted.

This study is not without limitations. Firstly, information on preexisting chronic conditions were collected based on patients' self-reports, and the renal dysfunction might have existed before the SARS-CoV-2 infection, but unknown to patients, which might influence our results. Secondly, the majority of patients received routine urine test at hospital admission, but not after then, so we could not differentiate the glomerular and tubular injuries caused by COVID-19. Thirdly, due to the follow-up duration of <3 months, we were unable to assess whether patients developed chronic kidney dysfunction due to COVID-19. Long-term follow-up studies are necessary to assess the occurrence rate of CKD and its outcomes.

In conclusion, COVID-19 patients can be at risk of AKI and continuous eGFR decline. Age ≥60 years, P/F ratio <300, and a higher creatinine at baseline independently predict the risk of AKI. The results from this study imply that it is necessary to evaluate and monitor the renal function of COVID-19 patients, especially for those who had renal dysfunction at baseline. Furthermore, our results suggest that it is necessary to continue to monitor renal function for those who still had abnormal eGFRs even when finally discharged from the hospital.

## Data Availability Statement

The raw data supporting the conclusions of this article will be made available by the authors, without undue reservation.

## Ethics Statement

The studies involving human participants were reviewed and approved by the Institutional Review Board of Shenzhen Third people's Hospital. Written informed consent to participate in this study was provided by the participants' legal guardian/next of kin.

## Author Contributions

JL and TW conceived the study and contributed equally. JC and F-SW supervised the study. JC organized the ethics approval and had full access to all of the data in the study. TW designed the data analysis pipeline and wrote the codes. JL run the codes on the data. JL, TW, and QH interpreted the data. JL, QC, DH, and LS collected clinical data. JL and TW wrote the manuscript. All the authors critically read, edited, and approved the manuscript.

## Conflict of Interest

The authors declare that the research was conducted in the absence of any commercial or financial relationships that could be construed as a potential conflict of interest.
