# Supplementary material for: Acute Kidney Injury and Early Predictive Factors in COVID-19 Patients
Source: Front Med (Lausanne). 2021 Jul 12;8:604242. doi: 10.3389/fmed.2021.604242 (PMC8311118; doi:10.3389/fmed.2021.604242)
Supplement: Supplementary file 1 [file Data_Sheet_1.docx]

**Supplementary Tables**

Table S1. Long-term medicines uses due to comorbidities before admission to the hospital

| Medicine use | Number of patients | Proportion |
| --- | --- | --- |
| None | 343 | 84.07 |
| Antihypertensive | 34 | 8.33 |
| Hypoglycemic | 9 | 2.21 |
| Antihypertensive+hypoglycemic | 5 | 1.23 |
| Antihypertensive+anticoagulants | 3 | 0.74 |
| Antihypertensive+antihyperlipidemic | 3 | 0.74 |
| Entecavir | 2 | 0.49 |
| Steroids | 2 | 0.49 |
| Hypoglycemic+anticoagulants | 1 | 0.25 |
| Brigatinib | 1 | 0.25 |
| Antiepileptic | 1 | 0.25 |
| Antihypertensive+antianxiety | 1 | 0.25 |
| Psychotropic | 1 | 0.25 |
| Antihypertensive+steroids | 1 | 0.25 |
| Anti-hyperuricemia | 1 | 0.25 |
| Total | 408 | 100.00 |

Table S2. Urine tests results at admission to the hospital^†^

| Urine | n(%) |
| --- | --- |
| n | 389(100) |
| Protein |  |
| negative or trace | 328(84.32) |
| 1+ | 53(13.62) |
| 2+ | 6(1.54) |
| 3+ | 2(0.51) |
| Blood |  |
| negative or trace | 348(89.46) |
| 1+ | 23(5.91) |
| 2+ | 16(4.11) |
| 3+ | 1(0.26) |

^†^19 patients did not receive urine test during hospitalization.

Table S3. The total proportions of AKI occurrence grouped by medicines use during hospitalization

| **Medicines** | **Patients Without AKI** | **Patients With AKI** | **Proportions** | **p values** |
| --- | --- | --- | --- | --- |
| **Total** | **392** | **16** | **16/408 = 3.9%** |  |
| Steroids |  |  |  |  |
| No | 292 | 6 | 6/298 = 2.0% | **0.003^*^** |
| **Yes** | **100** | **10** | **10/110 = 9.1%** |  |
| Antipyretic analgesics |  |  |  |  |
| No | 234 | 7 | 7/241=2.9% | 0.204 |
| **Yes** | **158** | **9** | **9/167=5.4%** |  |
| Antibiotic |  |  |  |  |
| No | 264 | 4 | 4/268=1.5% | **<0.001** |
| **Yes** | **128** | **12** | **12/140=8.6%** |  |
| Oseltamivir |  |  |  |  |
| No | 289 | 14 | 14/303=4.6% | 0.380**^*^** |
| **Yes** | **103** | **2** | **2/105=1.9%** |  |
| Interferon |  |  |  |  |
| No | 50 | 0 | 0/50=0% | 0.238^*^ |
| **Yes** | **342** | **16** | **16/358=4.5%** |  |
| Ritonavir-boosted lopinavir |  |  |  |  |
| No | 64 | 1 | 1/65=1.5% | 0.486 |
| **Yes** | 328 | 15 | 15/343=4.4% |  |

* Fisher's exact test

Table S4. Patients who experienced AKI during follow-up in each category of the lowest eGFR

| Category of the lowest eGFR | Patients experienced AKI |
| --- | --- |
| G1: >=90 ml/min/1.73 m^2^ | 2/283 |
| G2: 60-89 ml/min/1.73 m^2^ | 5/102 |
| G3a: 45-59 ml/min/1.73 m^2^ | 3/16 |
| G3b: 30-44 ml/min/1.73 m^2^ | 2/2 |
| G4: 15-29 ml/min/1.73 m^2^ | 3/4 |
| G5: <15 ml/min/1.73 m^2^ | 1/1 |

Table S5. Detailed information of renal function for the 43 patients who readmitted to the hospital

| Renal function | | At first admission | At first discharge | At re-admission | At second discharge |
| --- | --- | --- | --- | --- | --- |
| Normal renal function | eGFR ≥ 90 ml/min/1.73 m^2^ | 34/43 (79.1%) | 35/43 (81.4%) | 37/43 (86.0%) | 40/43 (93.0%) |
| Abnormal renal function | G2: 60-89 ml/min/1.73 m^2^ | 6/43 (13.9%) | 5/43 (11.6%) | 3/43 (7.0%) | 2/43 (4.7%) |
|  | G3a: 45-59 ml/min/1.73 m^2^ | 2/43 (4.7%) | 1/43 (2.3%) | 2/43 (4.7%) | 0 |
|  | G3b: 30-44 ml/min/1.73 m^2^ | 1/43 (2.3%) | 2/43 (4.7%) | 1/43 (2.3%) | 1/43 (2.3%) |
|  | G4: 15-29 ml/min/1.73 m^2^ | 0 | 0 | 0 | 0 |
|  | G5: <15 ml/min/1.73 m^2^ | 0 | 0 | 0 | 0 |
